# Uterine natural killer cells: Time for a re-appraisal?

**DOI:** 10.12688/f1000research.19132.1

**Published:** 2019-07-02

**Authors:** Judith N. Bulmer, Gendie E. Lash

**Affiliations:** 1 Institute of Cellular Medicine, Newcastle University, William Leech Building, Medical School, Framlington Place, Newcastle upon Tyne, NE2 4HH, UK; 2 Guangzhou Institute of Pediatrics, Guangzhou Women and Children’s Medical Center, 9 Jinsui Road, Guangzhou, Guangdong, 510623, China

**Keywords:** uterine natural killer cell, uNK cell, human pregnancy, decidua

## Abstract

The presence of unusual natural killer cells in human endometrium has been recognized for 30 years, but despite considerable research effort, the *in vivo* role of uterine natural killer (uNK) cells in both normal and pathological pregnancy remains uncertain. uNK cells may differentiate from precursors present in endometrium, but migration from peripheral blood in response to chemokine stimuli with *in situ* modification to a uNK cell phenotype is also possible. uNK cells produce a wide range of secretory products with diverse effects on trophoblast and spiral arteries which may play an important role in implantation and early placentation. Interactions with other decidual cell populations are also becoming clear. Recent evidence has demonstrated subpopulations of uNK cells and the presence of other innate lymphoid cell populations in decidua which may refine future approaches to investigation of the role of uNK cells in human pregnancy.

## Introduction

Granulated cells were recognized in endometrial stroma almost a century ago, but despite light and electron microscopic evidence for a lymphocyte origin in humans ^1^, mouse ^2, 3^, and rat ^4^, they were regarded as stromal cells until identified as unusual natural killer (NK) cells using monoclonal antibodies ^5– 7^. The term uterine NK (uNK) cell has been commonly adopted, although “decidual NK cell” and “endometrial NK cell” are also used to reflect their presence only in endometrium. uNK cells, characterized as CD45 ^+^CD56 ^bright^CD16 ^+^CD9 ^+^, increase in number in luteal phase endometrium and early pregnancy decidua ^7– 10^. A substantial number remain in later pregnancy ^11, 12^, although fewer have cytoplasmic granules, explaining why early studies relying on identifying cytoplasmic granules reported their virtual absence at full term ^13^. This distribution suggests a role in pregnancy and this has been the focus of studies over the last 30 years.

Despite suggestions of a detrimental role of NK cells in early pregnancy failure, uNK cells are considered to be a positive force for healthy pregnancy, although their precise role remains uncertain. The recent identification of molecularly distinct uNK cell subgroups ^14^ and other innate lymphoid cells (ILCs) in human decidua ^15– 17^ suggests that a re-appraisal may be timely. The aim of this review is to provide a brief overview of views on the origin and function of uNK cells that have developed over the last 30 years and consider future directions.

## Origin of uterine natural killer cells

There is no consensus regarding the origin of uNK cells; their gene expression patterns differ from those of peripheral blood NK (pbNK) cells ^18^, but whether they differentiate locally or are recruited to endometrium (or both) is uncertain. Hematopoietic precursor cells (HPCs) have been reported in non-pregnant endometrium ^19^ and early pregnancy decidua ^20– 22^ at a frequency of 0.1% to 4%. HPCs purified from decidua and cultured in decidual stromal cell (DSC)-conditioned medium or various cytokine combinations produced CD56 ^bright^CD16 ^−^CD9 ^+ ^uNK-like cells ^20– 22^. Additional evidence for local differentiation comes from detection of increased CD56 ^+ ^uNK cells in human proliferative endometrium transplanted into non-obese diabetic/severe combined immunodeficiency/γC ^null ^immunodeficient mice subjected to menstrual cycle–mimicking hormone treatment ^23^. In contrast, Male *et al*. ^24^ did not detect HPCs in non-pregnant endometrium and suggested the possibility of local differentiation from stage 3 immature NK cells, although the cells detected in these studies express markers that are characteristic oftype 3 ILCs and produce interleukin-22 (IL-22) ^17^. Human endometrium produces several factors implicated in NK cell differentiation, including IL-15 ^25^, IL-7, Flt3L ^26^, SCF (KL) ^27– 29^, and transforming growth factor beta 1 (TGFβ1) ^30^, many of which are increased in the luteal phase and early pregnancy.

Rather than differentiation from HPCs, it is possible that locally secreted chemokines/cytokines could attract pbNK cells to endometrium and also mediate further local differentiation. Purified CD16 ^+^CD9 ^− ^pbNK cells converted into CD16 ^−^CD9 ^+ ^uNK-like cells after culture with DSC-conditioned medium or TGFβ1 ^20, 31^, and a uNK cell-type phenotype (CD56 ^bright^CD16 ^−^CD9 ^+^KIR ^+^, VEGF-A producing, low cytotoxicity) was induced by exposure of pbNK cells to hypoxia, TGFβ1, and demethylating agents ^31^. DSCs produce chemoattractants implicated in recruitment of pbNK cells to endometrium, including C-X-C motif chemokine 10 (CXCL10), CXCL12, CX3CL1, and chemerin ^32, 33^, and compared with non-pregnant and male cells, pbNK cells from pregnant women have increased ability to migrate through DSCs ^26^. Furthermore, pbNK cells acquire a uNK cell-type chemokine receptor pattern after co-culture with DSCs. NK cell populations could also be recruited into endometrium in pregnancy in response to trophoblast ^34^.

uNK cells consistently localize to areas of stromal decidualization, including progesterone-treated endometrium, intrauterine decidua in ectopic pregnancy, and extrauterine decidua in normal pregnancy. This indicates a role for DSCs in uNK cell differentiation or recruitment (or both) and clearly demonstrates that they are not (all) dependent on trophoblast. It seems likely that both recruitment from blood and local differentiation play a role but perhaps with different contributions in non-pregnant and pregnant endometrium. A developing population is suggested by phenotypic differences with increasing gestation, and there is reduced expression of HLA-C–specific killer immunoglobulin-like receptors (KIRs) from 6 to 12 weeks ^35^, increased NKG2D expression from 8 to 12 weeks ^36^, increased expression of NKp80 and NKG2D, and a population with reduced CD56 brightness in second (13 to 20 weeks) compared with first (6 to 12 weeks) trimester ^11, 37^. Functional changes may also reflect an evolving population. Altered cytokine/growth factor expression and differing effects on trophoblast invasion have been reported in CD56 ^+^ uNK cells from 8 to 10 compared with 12 to 14 weeks of gestational age ^38– 40^, and a reduced proportion of cells expressing granzyme and perforin ^13, 37^, reduced CD56 ^+^ cells in proximity to extravillous trophoblast (EVT), and altered interactions with HTR-8/SVneo trophoblast-like cells have been reported in the second compared with the first trimester of pregnancy ^37^. These phenotypic and functional variations may reflect further local differentiation of immature NK cells or recruitment and further differentiation of additional populations from peripheral blood. CD56 ^+ ^cells also proliferate in endometrium, and the highest expression of the proliferation marker Ki67 (>40%) is in the mid/late luteal phase ^41^ and this could account for phenotypic differences as the menstrual cycle and pregnancy progress. The biological significance of the phenotypic and functional changes related to gestational age is not known. In addition, whether any altered decidual NK cell populations in pathological pregnancy represent alteration of a resident uNK cell population or recruitment of an additional population has not been established.

## Interaction with trophoblast

uNK cells express a range of receptors, including KIRs and leukocyte immunoglobulin-like receptors (LILRs) that can recognize HLA-E, HLA-G, and HLA-C, which are expressed by EVT ^42– 45^. uNK cell expression of these receptors differs from that of pbNK cells and is biased toward HLA-E and HLA-C1 recognition ^35^. It was suggested that the bias toward HLA-C recognition resulted from pregnancy ^46^, but studies using uNK cells from menstrual blood indicate that this receptor repertoire is established in non-pregnant endometrium and does not differ between consecutive menstrual cycles ^47, 48^. Expression of these specific receptors by uNK cells suggests that they interact with EVT, and specific KIR/HLA-C combinations have been associated with pregnancy complications, including pre-eclampsia, fetal growth restriction, and recurrent miscarriage ^49– 52^. However, an epidemiological study of Japanese couples did not support this proposal, noting similar rates of pre-eclampsia in couples comprising Japanese women and Caucasian men and couples comprising Japanese men and women ^53^. Furthermore, no association of HLA-C/KIR genotype with pre-eclampsia was noted in a recent Danish study, although this was confined to severe pre-eclampsia ^54^.

There were early suggestions that uNK cells may limit EVT invasion by cytotoxicity, and some studies have suggested that uNK cells are capable of cytotoxic activity. Co-culture of IL-2–activated CD56 ^bright^ decidual NK cells with an EVT-like cell line HTR-8/SV40neo led to granulolysin accumulation in the EVT cells, and transfection of granulolysin into HTR-8/SC40neo cells induced their apoptosis ^55^. Furthermore, a more recent study detected cytotoxic activity by IL-2–stimulated CD56 ^+^ cells (>90% purity) from early pregnancy decidua against both cytotrophoblast and the NK target K562, an activity that was inhibited by decidual macrophages ^56^. Nevertheless, in most studies, uNK cells have been poorly cytotoxic to both classic NK cell targets and trophoblast ^57^, and the consensus is that uNK cells are not cytotoxic in healthy pregnancy. uNK cells can acquire cytotoxic ability when decidua is infected by pathogens such as cytomegalovirus and toxoplasma, suggesting a possible role in protection against infection ^58^.

There are many reports of cytokine, chemokine, and growth factor production by uNK cells, mainly at a gestational age of 8 to 14 weeks, when trophoblast invasion and spiral artery remodeling are maximal; a summary is given in Table 1 but the list is not exhaustive. Several different approaches have been used for tissue disaggregation, uNK cell purification, and mechanisms and durations of cell activation; this may explain the variation between different studies.

**Table 1.** Cytokines, chemokines, and growth factor production by uterine natural killer cells.

Publication	Cytokines, chemokines, and growth factors detected	Cell preparation	Gestational age	Conditions
El Costa *et al*. ^45^	IFNγ, TNFα, MIP-1α, MIP-1β, GM-CSF	MACS-negative selection, 96% CD3−CD56+	8–12 weeks	Engagement of NKp30 (but not NKp46)
Higuma-Myojo *et al*. ^79^	TGF, minor populations of CD56+ cells producing other cytokines	Ficoll, flow cytometry	6–12 weeks	Mononuclear cells stimulated with PMA/ionomycin/brefeldin 4 hours, flow cytometry
Lash *et al*. ^38^	Ang1, Ang2, PDGF-BB (low), KGF (low), ICAM-1 (low), VEGF-C, PlGF, TGFβ1	MACS-positive selection, >95% CD56+	8–10 weeks, 12–14 weeks	No stimulation, Fastquant, ELISA
Lash *et al*. ^39^	IL-1β, IL-4, IL-6, IL-8, IL-10, IL-13, GM-CSF, IFNγ, RANTES, TNFα	MACS-positive selection >95% CD56+	8–10 weeks, 12–14 weeks	No stimulation, Fastquant, ELISA
Hanna *et al*. ^66^	CXCL8 (IL-8), CXCL10 (IP-10), CCL5 (RANTES), CCL22, MIP-1α, MIP-1β, PlGF, VEGF-A, VEGF-B, VEGF-C	Flow cytometry cell sorting, >99% CD56 ^bright^ CD16−CD3−	First trimester	IL-15 72 hours
Sotnikova *et al*. ^62^	IFNγ	Dynabeads-positive selection	7–10 weeks	Incubation with autologous cytotrophoblast
Engert *et al*. ^80^	GRO, MCP1, I-309, RANTES, IL-8, IL-1β, EGF, VEGF, TPO, M-CSF, ENA, oncostatin M, IL-10, GROα, angiogenin, IL-1α, IL-4, IL-12p40p70, IFNγ, SCF, SDF-1, MCP-2 (detected in >50% samples)	MACS, >90% pure	7–8 weeks	No stimulation, protein array
Saito *et al*. ^81^	G-CSF, GM-CSF, M-CSF, TNFα, IFNγ, LIF	Flow cytometry sorting	7–9 weeks	No stimulation
Fraser *et al*. ^82^	CXCL16, HB-EGF, HGF, IL-1β, IL-8, TGFβ1, UPA, TIMP-1	MACS-positive selection, 93.6 ± 1.3% CD56 ^+^, 80 ± 4% viability after 24 hours	9.1–13.7 weeks	IL-15, SCF
Wallace *et al*. ^83^	IL-6, IL-8, CXCL10	MagCellect-negative selection, CD56+ 95.7 ± 0.92%, viability after 6 hours 84.6 ± 2.8%	8–14 weeks	IL-15, SCF
Wallace *et al*. ^84^	Angiogenin, sIL2-R, endostatin, PlGF, IL-1RA, MIG, MIP- 1α, MIP-1β, RANTES	MACS-positive selection, 93.6 ± 1.3% CD56+	9–14 weeks	IL-15, SCF
Wallace *et al*. ^85^	TNFα, CXCL10, IFNγ, IL-8, PlGF (mRNA)	MagCellect-negative selection, CD56+ 95.7 ± 0.92%, viability after 6 hours 84.6 ± 2.8%	9–14 weeks	IL-15, SCF
Kennedy *et al*. ^63^	GM-CSF, CCL3, CCL1, XCL1	Flow cytometry and intracellular cytokine detection; or MACS positive selection and ELISA	First trimester	Activation of KIR2DS4
Chen *et al*. ^86^	Angiogenin, bFGF, VEGF-A, VEGFR, IL-1α	MACS-positive selection, 91.3% CD16−CD56+	LH+7 non-pregnant endometrium	
Fu *et al*. ^65^	Pleiotrophin, osteopontin, osteoglycin	Ficoll, MACS-negative selection	First trimester	Co-culture with EVT

Ang, angiopoetin; bFGF, basic fibroblast growth factor; CCL, C-C motif chemokine; EGF, epidermal growth factor; ELISA, enzyme- linked immunosorbent assay; ENA, epithelial neutrophil-activating protein; EVT, extravillous trophoblast; G-CSF, granulocyte colony-stimulating factor; GM-CSF, granulocyte-macrophage colony-stimulating factor; GRO, growth-regulated oncogene; HB-EGF, heparin-binding epidermal growth factor; HGF, hepatocyte growth factor; ICAM-1, intercellular adhesion molecule 1; IFN, interferon; IL, interleukin; KGF, keratinocyte growth factor; LIF, leukocyte inhibitory factor; MACS, magnetic-activated cell sorting; MCP, monocyte chemotactic protein; M-CSF, macrophage colony-stimulating factor; MIG, monokine induced by interferon-gamma; MIP, macrophage inflammatory protein; PDGF-BB, platelet-derived growth factor beta; PlGF, placental growth factor; RANTES, regulated upon activation, normal T cell expressed, and secreted; SCF, stem cell factor; SDF-1, stromal cell-derived factor 1; sIL2-R, soluble interleukin-2 receptor; TGF, transforming growth factor; TIMP-1, tissue inhibitor of metalloproteinase 1; TNFα, tumor necrosis factor alpha; TPO, thrombopoietin; UPA, urokinase-type plasminogen activator; VEGF, vascular endothelial growth factor; VEGFR, vascular endothelial growth factor receptor.

Given the phenotypic changes, it is not surprising that the uNK cell secretome varies with gestational age. uNK cell production of IL-1β, IL-6, CXCL8, granulocyte-macrophage colony-stimulating factor (GM-CSF), and interferon-gamma (IFNγ) was increased at 12 to 14 weeks compared with 8 to 10 weeks of gestation ^39^, whereas angiopoetin 2 (Ang2) and vascular endothelial growth factor-C (VEGF-C) secretion were higher at the earlier gestational age ^38^. Increased cytokine levels at 12 to 14 weeks may tie in with reports that interactions between pbNK cells and soluble HLA-G induce a pro-inflammatory/pro-angiogenic senescence-associated secretory phenotype with increased secretion of, among others, IL-1β, IL-6, and CXCL8 ^59– 61^, although this has not been demonstrated for uNK cells. uNK cell secretory products are also altered by trophoblast; uNK cell secretion of Ang1, VEGF-C, IL-6, CXCL8, and TGFβ1 were reduced by co-culture with EVT and cytotrophoblast ^40^. In contrast, increased IFNγ mRNA was reported after incubation of uNK cells with cytotrophoblast ^62^. Interaction of HLA-C (expressed by EVT) with the activating receptors KIR2DS1 and KIR2DS4 expressed by uNK cells stimulates their production of cytokines, including GM-CSF, which can stimulate trophoblast invasion *in vitro* ^63, 64^. Furthermore, a recent study demonstrated that, when co-cultured with EVT, a subset of uNK cells (CD49a ^+^Eomes ^+^) secrete growth-promoting factors, including pleiotrophin and osteoglycin; this effect is stimulated by HLA-G/ILT2 crosstalk ^65^.

Among other chemokines and growth factors, uNK cells simulate trophoblast invasion by production of CXCL8 and CXCL10 ^66^, although decidual CD3 ^+^ T cells, which produce higher levels of CXCL8, did not stimulate EVT invasion ^67^. Other cytokines produced by uNK cells, such as tumor necrosis factor alpha (TNFα) and IFNγ, inhibit invasion of EVT cells by various mechanisms, including trophoblast apoptosis, inhibition of proliferation, and reduced matrix metalloprotease (MMP) production ^68, 69^. The effect of uNK cells on EVT invasion may also depend on gestational age; in keeping with increased production of cytokines such as CXCL8, uNK cell supernatants at 12 to 14 weeks of gestation stimulated trophoblast invasion, whereas those at 8 to 10 weeks had no effect ^70^. In contrast, others have reported reduced trophoblast migration from villous explants co-cultured with decidual CD56 ^+ ^cells, and the effect was mediated by IFNγ ^62, 71^.

uNK cells have a complex array of secretory products which may alter with different approaches to purification and activation. Despite the many and varied reports, the *in vivo* relevance of these studies remains unclear. Examination of any one cytokine is unlikely to reflect its importance *in vivo*, which may vary with gestational age; for example, a role in spiral artery remodeling may shift in later gestation to promoting trophoblast invasion. In regard to the role of uNK cells in the regulation of trophoblast invasion, it is important to remember that the cells are confined to decidua/endometrium, whereas even in the first trimester of normal pregnancy, interstitial trophoblast invades into inner myometrium ^72^. Trophoblast also invades into myometrium in placenta accreta spectrum disorders, in which decidua (and uNK cells) are absent ^73^. Therefore, other factors, including the inherent invasive capacity of EVT following epithelial–mesenchymal transition ^74, 75^ and other maternal cell populations, must play a role.

## Interaction with spiral arteries

Remodeling of spiral arteries in decidua basalis and underlying superficial myometrium is essential for healthy pregnancy and it is now accepted that, in addition to trophoblast, maternal uterine cells play a role ^76, 77^, although the presence of trophoblast may be required for the uNK cell effect ^78^. Morphological changes in spiral arteries and arterioles in luteal phase endometrium and intrauterine decidua in ectopic pregnancy also suggest a trophoblast-independent effect ^76^.

uNK cells from decidua of 8 to 10 weeks, but not 12 to 14 weeks, of gestation can induce separation of vascular smooth muscle cells and loss of extracellular matrix via secretion of Ang1, Ang2, VEGF-C, and IFNγ ^87^. uNK cells (and macrophages) also produce MMPs that can break down collagen in an *in vitro* spiral artery model ^88^. uNK cell supernatants also mediate vascular smooth muscle cell dedifferentiation and this effect is seen at 12 to 14 weeks but not 8 to 10 weeks of gestation ^89^. Other chemokines and growth factors have been implicated in the uNK cell effect on spiral arteries. For example, Choudhury *et al*. ^90^ suggested that IL-6 and CXCL8 secreted by EVT activate secretion of C-C motif chemokine 14 (CCL14) and CXCL6 by endothelial cells which then recruit uNK cells and macrophages to spiral arteries. Both IL-6 and CXCL8 also induce dedifferentiation of vascular smooth muscle cells in a chorionic plate artery model ^91^.

Altered decidual leucocytes have been reported in pre-eclampsia, although results have varied ^92– 94^; there have been reports of reduced ^95– 98^, unchanged ^99^, and increased ^100, 101^ uNK cells compared with controls. Uterine artery Doppler studies in early pregnancy can predict women at increased risk of developing pre-eclampsia in later pregnancy ^102^; compared with post-delivery samples, these high-risk samples allow investigation when the pathogenetic lesion develops, before compensatory measures are in place, and this approach has been used to investigate uNK cell function in pregnancies at high risk of developing pre-eclampsia. uNK cells from 9- to 14-week gestational age pregnancies with abnormal Doppler wave forms showed reduced expression of KIR2DL/S1,3,5 and LILRB1 ^85^, reduced stimulation of trophoblast outgrowth from villous explants ^83^, reduced *in vitro* induction of apoptosis in vascular smooth muscle cells and endothelial cells ^82^, increased angiogenic growth factor secretion ^84^, and reduced ability to destabilize endothelial structures ^103^. These results all suggest that uNK cells from pregnancies at increased risk of pre-eclampsia display altered effects on trophoblast invasion and spiral arteries and emphasize the likely importance of uNK cells in early pregnancy. Nevertheless, in common with trophoblast invasion, spiral artery remodeling extends into inner myometrium. CD14 ^+^ macrophages and CD3 ^+^ T cells are present in the wall and adventitia of spiral arteries in both decidua and superficial myometrium ^104^, suggesting that these cells also have a role, either alone or in collaboration with uNK cells.

## Interactions with other cells

uNK cells may also interact with other cell types in non-pregnant and pregnant endometrium. uNK cells interact with CD14 ^+^ cells in decidua to produce IDO (indoleamine-2,3-dioxygenase), which induces regulatory T cells ^105^. This interaction appears to be mediated by IFNγ and TGFβ and is not seen with pbNK cells or CD14 ^+^ cells. uNK cells also form conjugates with immature dendritic cells in first-trimester decidua ^106– 108^ which can induce uNK cell proliferation and cytotoxicity ^109^. It has also been suggested that uNK cells induce apoptosis of CD209 (DC-SIGN) ^+^ dendritic cells in decidua ^108^. Evidence from mouse pregnancy suggests that IL-10 secreted by uNK cells regulates dendritic cell phenotype and function, and IL-10 deficiency and dendritic cell expansion are associated with early pregnancy failure ^110^.

In addition to affecting their recruitment or differentiation (or both), DSCs may affect uNK cell function; cultured first-trimester DSCs inhibited proliferation, cytotoxicity, IFNγ production, and upregulation of activation receptor expression by pbNK cells ^111^. In non-pregnant endometrium, uNK cells may clear senescent decidual cells at the end of the menstrual cycle, playing a crucial role in endometrial homeostasis ^112^.

## Summary

Knowledge has increased dramatically since their recognition as unusual NK cells, but despite considerable research effort, the *in vivo* role of uNK cells remains unclear. Recent advances suggest that a re-appraisal may be timely. Whether uNK cell phenotype and function differ between non-pregnant and pregnant endometrium, between decidua basalis and parietalis, or at different sites within decidua (such as those related to spiral arteries) and at different gestational ages remains largely unknown. Pre-eclampsia, fetal growth restriction, and recurrent pregnancy failure have been associated with altered uNK cell numbers and function and specific KIR/HLA-C combinations but the significance of these observations is not fully established. uNK cells produce a wide range of chemokines, cytokines, growth factors, and MMPs, and many have been shown to have specific effects on trophoblast or spiral arteries, but *in vivo* translation is difficult. Gestational age differences in phenotype and function suggest that including samples across a range of gestational weeks in the first trimester may result in skewing of data.

The starting point for most studies of uNK cell function is decidua retrieved from pregnancy terminations or miscarriages. uNK cells within these samples have been exposed directly to EVT or to soluble HLA-G with possible functional effects. Investigations of uNK cells purified from non-pregnant endometrium are relatively infrequent, although several studies have reported increased uNK cells in luteal phase endometrium in recurrent early pregnancy failure ^113– 116^. A recent study of uNK cells from timed luteal phase endometrium reported increased expression of angiogenin, VEGF-A, and basic fibroblast growth factor (bFGF) in women with recurrent miscarriage compared with fertile controls ^86^. Recent studies indicate that menstrual blood may act as a surrogate for endometrial NK cells ^47, 48, 117^ and this approach may increase the scope for future studies.

A single-cell RNA-sequencing (scRNA-seq) study of first-trimester decidua recently defined three distinct uNK cell populations ^15^. The first (termed dNK1) contained more cytoplasmic granules, higher cytoplasmic granule proteins, and higher expression of KIR genes able to bind to HLA-C; LILRB1, which has high affinity for HLA-G, was expressed only by the dNK1 subset, suggesting that this subset interacts particularly with EVT. The second population (dNK2) also expressed activating NKG2C and NKG2E and inhibitory NKG2A receptors for HLA-E molecules, whereas the third population (dNK3) did not express these receptors but did express CCL5, suggesting a role in the regulation of EVT invasion via C-C motif chemokine receptor 1 (CCR1). This report demonstrates subpopulations of uNK cells which are likely to have diverse functions. Similar studies of pathological pregnancy and non-pregnant endometrium, including endometrial NK cell populations in women with recurrent early pregnancy failure, may clarify the roles of specific uNK cell subsets in pathological pregnancy. The suggestion that there are subsets of NK cells in human decidua is supported by a report of production of growth-promoting factors by CD49a ^+^Eomes ^+^ uNK cells via interactions with HLA-G ^65^, as well as the demonstration of a subpopulation of “pregnancy-trained” uNK cells in repeated compared with first pregnancies, characterized by high expression of the receptors NKG2C and LILRB1 and increased IFNγ and VEGF-A secretion ^118^.

Besides uNK cells, other innate lymphoid cells have been identified in human decidua ^15– 17^, including CD56 ^+^CD94 ^−^ non-cytotoxic type 1 ILCs (ILC1s), CD56 ^−^CD117 ^+^CD127 ^+^ lymphoid tissue inducer (LTi)-like cells, and CD56 ^+^CD94 ^−^CD117 ^+^CD127 ^+^NKp44 ^+^ type 3 ILCs (ILC3s). These ILCs may contribute to the cytokine production reported by decidual CD56 ^+^ cells. For example, ILC1s may contribute to IFNγ production, while ILC3s produce IL-22 and CXCL8, which may regulate neutrophil recruitment ^119^. The distribution and function of the different ILC populations are still unknown, although it has been suggested that ILC3 cytokine production may be regulated by programmed cell death (PD-1) expressed by ILC3s and its ligand PD-L1 expressed by EVT ^120^.

It is now clear that “uNK cells” are not a single population and the relative importance of these subpopulations may change as gestation progresses. ILCs in decidua may contribute to the reported cytokine production by CD56 ^+^ uNK cells and this may differ according to different purification and activation protocols. Abnormal function in pathological pregnancy could affect specific uNK cell subpopulations or ILCs. Rather than referring to “uNK cells”, perhaps we should dissect populations more precisely and consider the functional contribution of ILCs. Technical advances may allow localization of different subpopulations, and investigation of pathological pregnancies may provide valuable clues to function. The advent of scRNA-seq technology provides an exciting way forward to unravel the role of uNK cells in normal and pathological pregnancy, making it possible to target specific cell populations for more accurate diagnosis and potential intervention.

## References

[1] Weill P: Les cellules granuleuses des muqueuses intestinale et uterine. *Arch Anat Microsc.* 1921;17:77–82. Reference Source

[2] Smith LJ: Metrial gland and other glycogen containing cells in the mouse uterus following mating and through implantation of the embryo. *Am J Anat.* 1966;119(1):15–23. 10.1002/aja.1001190103 4165356

[3] Stewart I Peel S: The structure and differentiation of granulated metrial gland cells of the pregnant mouse uterus. *Cell Tissue Res.* 1977;184(4):517–27. 10.1007/BF00220975 589658

[4] Peel S Bulmer D: The fine structure of the rat metrial gland in relation to the origin of the granulated cells. *J Anat.* 1977;123(Pt 3):687–96. 885782 PMC1234726

[5] Ritson A Bulmer JN: Endometrial granulocytes in human decidua react with a natural-killer (NK) cell marker, NKH1. *Immunology.* 1987;62(2):329–31. 3315980 PMC1453990

[6] Starkey PM Sargent IL Redman CW: Cell populations in human early pregnancy decidua: characterization and isolation of large granular lymphocytes by flow cytometry. *Immunology.* 1988;65(1):129–34. 3181993 PMC1385031

[7] King A Wellings V Gardner L: Immunocytochemical characterization of the unusual large granular lymphocytes in human endometrium throughout the menstrual cycle. *Hum Immunol.* 1989;24(3):195–205. 10.1016/0198-8859(89)90060-8 2925453

[8] Bulmer JN Morrison L Longfellow M: Granulated lymphocytes in human endometrium: histochemical and immunohistochemical studies. *Hum Reprod.* 1991;6(6):791–8. 10.1093/oxfordjournals.humrep.a137430 1757516

[9] Marshall RJ Jones DB: An immunohistochemical study of lymphoid tissue in human endometrium. *Int J Gynecol Pathol.* 1988;7(3):225–35. 3182169 10.1097/00004347-198809000-00003

[10] Kamat BR Isaacson PG: The immunocytochemical distribution of leukocytic subpopulations in human endometrium. *Am J Pathol.* 1987;127(1):66–73. 3565538 PMC1899604

[11] Kwan M Hazan A Zhang J: Dynamic changes in maternal decidual leukocyte populations from first to second trimester gestation. *Placenta.* 2014;35(12):1027–34. 10.1016/j.placenta.2014.09.018 25449030

[12] Williams PJ Searle RF Robson SC: Decidual leucocyte populations in early to late gestation normal human pregnancy. *J Reprod Immunol.* 2009;82(1):24–31. 10.1016/j.jri.2009.08.001 19732959

[13] Bulmer JN Williams PJ Lash GE: Immune cells in the placental bed. *Int J Dev Biol.* 2010;54(2–3):281–94. 10.1387/ijdb.082763jb 19876837

[14] Vento-Tormo R Efremova M Botting RA: Single-cell reconstruction of the early maternal-fetal interface in humans. *Nature.* 2018;563(7731):347–53. 10.1038/s41586-018-0698-6 30429548 PMC7612850

[15] Vacca P Vitale C Munari E: Human Innate Lymphoid Cells: Their Functional and Cellular Interactions in Decidua. *Front Immunol.* 2018;9:1897. 10.3389/fimmu.2018.01897 30154799 PMC6102343

[16] Vacca P Montaldo E Croxatto D: Identification of diverse innate lymphoid cells in human decidua. *Mucosal Immunol.* 2015;8(2):254–64. 10.1038/mi.2014.63 25052762

[17] Miller D Motomura K Garcia-Flores V: Innate Lymphoid Cells in the Maternal and Fetal Compartments. *Front Immunol.* 2018;9:2396. 10.3389/fimmu.2018.02396 30416502 PMC6212529

[18] Koopman LA Kopcow HD Rybalov B: Human decidual natural killer cells are a unique NK cell subset with immunomodulatory potential. *J Exp Med.* 2003;198(8):1201–12. 10.1084/jem.20030305 14568979 PMC2194228

[19] Lynch L Golden-Mason L Eogan M: Cells with haematopoietic stem cell phenotype in adult human endometrium: relevance to infertility? *Hum Reprod.* 2007;22(4):919–26. 10.1093/humrep/del456 17208945

[20] Keskin DB Allan DS Rybalov B: TGFbeta promotes conversion of CD16 ^+^ peripheral blood NK cells into CD16 ^-^ NK cells with similarities to decidual NK cells. *Proc Natl Acad Sci U S A.* 2007;104(9):3378–83. 10.1073/pnas.0611098104 17360654 PMC1805591

[21] Vacca P Vitale C Montaldo E: CD34 ^+^ hematopoietic precursors are present in human decidua and differentiate into natural killer cells upon interaction with stromal cells. *Proc Natl Acad Sci U S A.* 2011;108(6):2402–7. 10.1073/pnas.1016257108 21248224 PMC3038730

[22] Szereday L Miko E Meggyes M: Commitment of decidual haematopoietic progenitor cells in first trimester pregnancy. *Am J Reprod Immunol.* 2012;67(1):9–16. 10.1111/j.1600-0897.2011.01029.x 21682790

[23] Matsuura-Sawada R Murakami T Ozawa Y: Reproduction of menstrual changes in transplanted human endometrial tissue in immunodeficient mice. *Hum Reprod.* 2005;20(6):1477–84. 10.1093/humrep/deh783 15734760

[24] Male V Hughes T McClory S: Immature NK cells, capable of producing IL-22, are present in human uterine mucosa. *J Immunol.* 2010;185(7):3913–8. 10.4049/jimmunol.1001637 20802153 PMC3795409

[25] Kitaya K Yasuda J Yagi I: IL-15 expression at human endometrium and decidua. *Biol Reprod.* 2000;63(3):683–7. 10.1095/biolreprod63.3.683 10952908

[26] Hannan NJ Paiva P Meehan KL: Analysis of fertility-related soluble mediators in human uterine fluid identifies VEGF as a key regulator of embryo implantation. *Endocrinology.* 2011;152(12):4948–56. 10.1210/en.2011-1248 22028446

[27] Sharkey AM Jokhi PP King A: Expression of c-kit and kit ligand at the human maternofetal interface. *Cytokine.* 1994;6(2):195–205. 10.1016/1043-4666(94)90042-6 7518262

[28] Umekage H Saito S Morikawa H: Enhancement by stem cell factor of interleukin-2 (IL-2)-induced DNA synthesis in human decidual CD16- CD56 ^bright^ natural killer cells mediated by increased expression of the IL-2 receptor alpha chain. *J Reprod Immunol.* 1998;40(1):1–24. 10.1016/S0165-0378(98)00028-X 9862254

[29] Kauma S Huff T Krystal G: The expression of stem cell factor and its receptor, c-kit in human endometrium and placental tissues during pregnancy. *J Clin Endocrinol Metab.* 1996;81(3):1261–6. 10.1210/jcem.81.3.8772609 8772609

[30] Jones RL Stoikos C Findlay JK: TGF-beta superfamily expression and actions in the endometrium and placenta. *Reproduction.* 2006;132(2):217–32. 10.1530/rep.1.01076 16885531

[31] Cerdeira AS Rajakumar A Royle CM: Conversion of peripheral blood NK cells to a decidual NK-like phenotype by a cocktail of defined factors. *J Immunol.* 2013;190(8):3939–48. 10.4049/jimmunol.1202582 23487420 PMC3742368

[32] Carlino C Stabile H Morrone S: Recruitment of circulating NK cells through decidual tissues: a possible mechanism controlling NK cell accumulation in the uterus during early pregnancy. *Blood.* 2008;111(6):3108–15. 10.1182/blood-2007-08-105965 18187664

[33] Carlino C Trotta E Stabile H: Chemerin regulates NK cell accumulation and endothelial cell morphogenesis in the decidua during early pregnancy. *J Clin Endocrinol Metab.* 2012;97(10):3603–12. 10.1210/jc.2012-1102 22791765 PMC3462933

[34] Wu X Jin LP Yuan MM: Human first-trimester trophoblast cells recruit CD56 ^bright^CD16 ^-^ NK cells into decidua by way of expressing and secreting of CXCL12/stromal cell-derived factor 1. *J Immunol.* 2005;175(1):61–8. 10.4049/jimmunol.175.1.61 15972632

[35] Sharkey AM Gardner L Hiby S: Killer Ig-like receptor expression in uterine NK cells is biased toward recognition of HLA-C and alters with gestational age. *J Immunol.* 2008;181(1):39–46. 10.4049/jimmunol.181.1.39 18566368

[36] Marlin R Duriez M Berkane N: Dynamic shift from CD85j/ILT-2 to NKG2D NK receptor expression pattern on human decidual NK during the first trimester of pregnancy. *PLoS One.* 2012;7(1):e30017. 10.1371/journal.pone.0030017 22242197 PMC3252358

[37] Zhang J Dunk CE Kwan M: Human dNK cell function is differentially regulated by extrinsic cellular engagement and intrinsic activating receptors in first and second trimester pregnancy. *Cell Mol Immunol.* 2017;14(2):203–13. 10.1038/cmi.2015.66 26277900 PMC5301153

[38] Lash GE Schiessl B Kirkley M: Expression of angiogenic growth factors by uterine natural killer cells during early pregnancy. *J Leukoc Biol.* 2006;80(3):572–80. 10.1189/jlb.0406250 16816146

[39] Lash GE Robson SC Bulmer JN: Review: Functional role of uterine natural killer (uNK) cells in human early pregnancy decidua. *Placenta.* 2010;31 Suppl:S87–S92. 10.1016/j.placenta.2009.12.022 20061017

[40] Lash GE Naruse K Robson A: Interaction between uterine natural killer cells and extravillous trophoblast cells: effect on cytokine and angiogenic growth factor production. *Hum Reprod.* 2011;26(9):2289–95. 10.1093/humrep/der198 21685139

[41] Jones RK Searle RF Stewart JA: Apoptosis, *bcl*-2 expression, and proliferative activity in human endometrial stroma and endometrial granulated lymphocytes. *Biol Reprod.* 1998;58(4):995–1002. 10.1095/biolreprod58.4.995 9546731

[42] Chazara O Xiong S Moffett A: Maternal KIR and fetal HLA-C: a fine balance. *J Leukoc Biol.* 2011;90(4):703–16. 10.1189/jlb.0511227 21873457

[43] Apps R Gardner L Sharkey AM: A homodimeric complex of HLA-G on normal trophoblast cells modulates antigen-presenting cells *via* LILRB1. *Eur J Immunol.* 2007;37(7):1924–37. 10.1002/eji.200737089 17549736 PMC2699429

[44] Apps R Sharkey A Gardner L: *Ex vivo* functional responses to HLA-G differ between blood and decidual NK cells. *Mol Hum Reprod.* 2011;17(9):577–86. 10.1093/molehr/gar022 21471023 PMC3160205

[45] El Costa H Casemayou A Aguerre-Girr M: Critical and differential roles of nkp46- and nkp30-activating receptors expressed by uterine NK cells in early pregnancy. *J Immunol.* 2008;181(5):3009–17. 10.4049/jimmunol.181.5.3009 18713971

[46] Male V Sharkey A Masters L: The effect of pregnancy on the uterine NK cell KIR repertoire. *Eur J Immunol.* 2011;41(10):3017–27. 10.1002/eji.201141445 21739430 PMC3262970

[47] Ivarsson MA Stiglund N Marquardt N: Composition and dynamics of the uterine NK cell KIR repertoire in menstrual blood. *Mucosal Immunol.* 2017;10(2):322–31. 10.1038/mi.2016.50 27271316

[48] Feyaerts D Kuret T van Cranenbroek B: Endometrial natural killer (NK) cells reveal a tissue-specific receptor repertoire. *Hum Reprod.* 2018;33(3):441–51. 10.1093/humrep/dey001 29447367

[49] Hiby SE Walker JJ O'Shaughnessy KM: Combinations of maternal KIR and fetal HLA-C genes influence the risk of preeclampsia and reproductive success. *J Exp Med.* 2004;200(8):957–65. 10.1084/jem.20041214 15477349 PMC2211839

[50] Hiby SE Regan L Lo W: Association of maternal killer-cell immunoglobulin-like receptors and parental HLA-C genotypes with recurrent miscarriage. *Hum Reprod.* 2008;23(4):972–6. 10.1093/humrep/den011 18263639

[51] Hiby SE Apps R Sharkey AM: Maternal activating KIRs protect against human reproductive failure mediated by fetal HLA-C2. *J Clin Invest.* 2010;120(11):4102–10. 10.1172/JCI43998 20972337 PMC2964995

[52] Huhn O Chazara O Ivarsson MA: High-Resolution Genetic and Phenotypic Analysis of KIR2DL1 Alleles and Their Association with Pre-Eclampsia. *J Immunol.* 2018;201(9):2593–601. 10.4049/jimmunol.1800860 30249807 PMC6258046

[53] Saito S Takeda Y Sakai M: The incidence of pre-eclampsia among couples consisting of Japanese women and Caucasian men. *J Reprod Immunol.* 2006;70(1–2):93–8. 10.1016/j.jri.2005.12.005 16427138

[54] Larsen TG Hackmon R Geraghty DE: Fetal human leukocyte antigen-C and maternal killer-cell immunoglobulin-like receptors in cases of severe preeclampsia. *Placenta.* 2019;75:27–33. 10.1016/j.placenta.2018.11.008 30712663

[55] Nakashima A Shiozaki A Myojo S: Granulysin produced by uterine natural killer cells induces apoptosis of extravillous trophoblasts in spontaneous abortion. *Am J Pathol.* 2008;173(3):653–64. 10.2353/ajpath.2008.071169 18688023 PMC2527087

[56] Co EC Gormley M Kapidzic M: Maternal decidual macrophages inhibit nk cell killing of invasive cytotrophoblasts during human pregnancy. *Biol Reprod.* 2013;88(6):155. 10.1095/biolreprod.112.099465 23553431 PMC4070869

[57] Kopcow HD Allan DS Chen X: Human decidual NK cells form immature activating synapses and are not cytotoxic. *Proc Natl Acad Sci U S A.* 2005;102(43):15563–8. 10.1073/pnas.0507835102 16230631 PMC1266146

[58] Le Bouteiller P Bensussan A: Up-and-down immunity of pregnancy in humans [version 1; peer review: 2 approved]. *F1000Res.* 2017;6:1216. 10.12688/f1000research.11690.1 28781765 PMC5531158

[59] Rajagopalan S Bryceson YT Kuppusamy SP: Activation of NK cells by an endocytosed receptor for soluble HLA-G. *PLoS Biol.* 2006;4(1):e9. 10.1371/journal.pbio.0040009 16366734 PMC1318474

[60] Rajagopalan S Long EO: Cellular senescence induced by CD158d reprograms natural killer cells to promote vascular remodeling. *Proc Natl Acad Sci U S A.* 2012;109(50):20596–601. 10.1073/pnas.1208248109 23184984 PMC3528503

[61] Rajagopalan S: HLA-G-mediated NK cell senescence promotes vascular remodeling: implications for reproduction. *Cell Mol Immunol.* 2014;11(5):460–6. 10.1038/cmi.2014.53 24998350 PMC4197210

[62] Sotnikova N Voronin D Antsiferova Y: Interaction of decidual CD56+ NK with trophoblast cells during normal pregnancy and recurrent spontaneous abortion at early term of gestation. *Scand J Immunol.* 2014;80(3):198–208. 10.1111/sji.12196 24909810

[63] Kennedy PR Chazara O Gardner L: Activating KIR2DS4 Is Expressed by Uterine NK Cells and Contributes to Successful Pregnancy. *J Immunol.* 2016;197(11):4292–300. 10.4049/jimmunol.1601279 27815424 PMC5114884

[64] Xiong S Sharkey AM Kennedy PR: Maternal uterine NK cell-activating receptor KIR2DS1 enhances placentation. *J Clin Invest.* 2013;123(10):4264–72. 10.1172/JCI68991 24091323 PMC4382274

[65] Fu B Zhou Y Ni X: Natural Killer Cells Promote Fetal Development through the Secretion of Growth-Promoting Factors. *Immunity.* 2017;47(6):1100–1113.e6. 10.1016/j.immuni.2017.11.018 29262349

[66] Hanna J Goldman-Wohl D Hamani Y: Decidual NK cells regulate key developmental processes at the human fetal-maternal interface. *Nat Med.* 2006;12(9):1065–74. 10.1038/nm1452 16892062

[67] De Oliveira LG Lash GE Murray-Dunning C: Role of interleukin 8 in uterine natural killer cell regulation of extravillous trophoblast cell invasion. *Placenta.* 2010;31(7):595–601. 10.1016/j.placenta.2010.04.012 20483454

[68] Lash GE Otun HA Innes BA: Interferon-gamma inhibits extravillous trophoblast cell invasion by a mechanism that involves both changes in apoptosis and protease levels. *FASEB J.* 2006;20(14):2512–8. 10.1096/fj.06-6616com 17142800

[69] Otun HA Lash GE Innes BA: Effect of tumour necrosis factor-α in combination with interferon-γ on first trimester extravillous trophoblast invasion. *J Reprod Immunol.* 2011;88(1):1–11. 10.1016/j.jri.2010.10.003 21112094

[70] Lash GE Otun HA Innes BA: Regulation of extravillous trophoblast invasion by uterine natural killer cells is dependent on gestational age. *Hum Reprod.* 2010;25(5):1137–45. 10.1093/humrep/deq050 20219775

[71] Hu Y Dutz JP MacCalman CD: Decidual NK cells alter *in vitro* first trimester extravillous cytotrophoblast migration: a role for IFN-gamma. *J Immunol.* 2006;177(12):8522–30. 10.4049/jimmunol.177.12.8522 17142750

[72] Pijnenborg R Vercruysse L Hanssens M: The uterine spiral arteries in human pregnancy: facts and controversies. *Placenta.* 2006;27(9–10):939–58. 10.1016/j.placenta.2005.12.006 16490251

[73] Hannon T Innes BA Lash GE: Effects of local decidua on trophoblast invasion and spiral artery remodeling in focal placenta creta - an immunohistochemical study. *Placenta.* 2012;33(12):998–1004. 10.1016/j.placenta.2012.09.004 23040667

[74] DaSilva-Arnold S James JL Al-Khan A: Differentiation of first trimester cytotrophoblast to extravillous trophoblast involves an epithelial-mesenchymal transition. *Placenta.* 2015;36(12):1412–8. 10.1016/j.placenta.2015.10.013 26545962

[75] E Davies J Pollheimer J Yong HE: Epithelial-mesenchymal transition during extravillous trophoblast differentiation. *Cell Adh Migr.* 2016;10(3):310–21. 10.1080/19336918.2016.1170258 27070187 PMC4951171

[76] Craven CM Morgan T Ward K: Decidual spiral artery remodelling begins before cellular interaction with cytotrophoblasts. *Placenta.* 1998;19(4):241–52. 10.1016/S0143-4004(98)90055-8 9639319

[77] Smith SD Dunk CE Aplin JD: Evidence for immune cell involvement in decidual spiral arteriole remodeling in early human pregnancy. *Am J Pathol.* 2009;174(5):1959–71. 10.2353/ajpath.2009.080995 19349361 PMC2671283

[78] Hazan AD Smith SD Jones RL: Vascular-leukocyte interactions: mechanisms of human decidual spiral artery remodeling *in vitro*. *Am J Pathol.* 2010;177(2):1017–30. 10.2353/ajpath.2010.091105 20558572 PMC2913364

[79] Higuma-Myojo S Sasaki Y Miyazaki S: Cytokine profile of natural killer cells in early human pregnancy. *Am J Reprod Immunol.* 2005;54(1):21–9. 10.1111/j.1600-0897.2005.00279.x 15948769

[80] Engert S Rieger L Kapp M: Profiling chemokines, cytokines and growth factors in human early pregnancy decidua by protein array. *Am J Reprod Immunol.* 2007;58(2):129–37. 10.1111/j.1600-0897.2007.00498.x 17631006

[81] Saito S Nishikawa K Morii T: Cytokine production by CD16-CD56 ^bright^ natural killer cells in the human early pregnancy decidua. *Int Immunol.* 1993;5(5):559–63. 10.1093/intimm/5.5.559 7686393

[82] Fraser R Whitley GS Johnstone AP: Impaired decidual natural killer cell regulation of vascular remodelling in early human pregnancies with high uterine artery resistance. *J Pathol.* 2012;228(3):322–32. 10.1002/path.4057 22653829 PMC3499663

[83] Wallace AE Host AJ Whitley GS: Decidual natural killer cell interactions with trophoblasts are impaired in pregnancies at increased risk of preeclampsia. *Am J Pathol.* 2013;183(6):1853–61. 10.1016/j.ajpath.2013.08.023 24103555 PMC4188218

[84] Wallace AE Fraser R Gurung S: Increased angiogenic factor secretion by decidual natural killer cells from pregnancies with high uterine artery resistance alters trophoblast function. *Hum Reprod.* 2014;29(4):652–60. 10.1093/humrep/deu017 24522839 PMC3949498

[85] Wallace AE Whitley GS Thilaganathan B: Decidual natural killer cell receptor expression is altered in pregnancies with impaired vascular remodeling and a higher risk of pre-eclampsia. *J Leukoc Biol.* 2015;97(1):79–86. 10.1189/jlb.2A0614-282R 25381387 PMC4377829

[86] Chen X Liu Y Cheung WC: Increased expression of angiogenic cytokines in CD56+ uterine natural killer cells from women with recurrent miscarriage. *Cytokine.* 2018;110:272–6. 10.1016/j.cyto.2018.01.013 29396049

[87] Robson A Harris LK Innes BA: Uterine natural killer cells initiate spiral artery remodeling in human pregnancy. *FASEB J.* 2012;26(12):4876–85. 10.1096/fj.12-210310 22919072

[88] Choudhury RH Dunk CE Lye SJ: Decidual leucocytes infiltrating human spiral arterioles are rich source of matrix metalloproteinases and degrade extracellular matrix *in vitro* and *in situ*. *Am J Reprod Immunol.* 2019;81(1):e13054. 10.1111/aji.13054 30267451

[89] Robson A Lash GE Innes BA: Uterine natural killer cells mediate dedifferentiation of spiral artery vascular smooth muscle cells in early human pregnancy. *Hum Reprod.*in press.

[90] Choudhury RH Dunk CE Lye SJ: Extravillous Trophoblast and Endothelial Cell Crosstalk Mediates Leukocyte Infiltration to the Early Remodeling Decidual Spiral Arteriole Wall. *J Immunol.* 2017;198(10):4115–28. 10.4049/jimmunol.1601175 28396316

[91] Pitman H Innes BA Robson SC: Altered expression of interleukin-6, interleukin-8 and their receptors in decidua of women with sporadic miscarriage. *Hum Reprod.* 2013;28(8):2075–86. 10.1093/humrep/det233 23739222

[92] Cartwright JE James-Allan L Buckley RJ: The role of decidual NK cells in pregnancies with impaired vascular remodelling. *J Reprod Immunol.* 2017;119:81–4. 10.1016/j.jri.2016.09.002 27680579

[93] Faas MM De Vos P: Innate immune cells in the placental bed in healthy pregnancy and preeclampsia. *Placenta.* 2018;69:125–33. 10.1016/j.placenta.2018.04.012 29748088

[94] Hsu P Nanan RK: Innate and adaptive immune interactions at the fetal-maternal interface in healthy human pregnancy and pre-eclampsia. *Front Immunol.* 2014;5:125. 10.3389/fimmu.2014.00125 24734032 PMC3975095

[95] Williams PJ Bulmer JN Searle RF: Altered decidual leucocyte populations in the placental bed in pre-eclampsia and foetal growth restriction: a comparison with late normal pregnancy. *Reproduction.* 2009;138(1):177–84. 10.1530/REP-09-0007 19357130

[96] Rieger L Segerer S Bernar T: Specific subsets of immune cells in human decidua differ between normal pregnancy and preeclampsia--a prospective observational study. *Reprod Biol Endocrinol.* 2009;7:132. 10.1186/1477-7827-7-132 19930648 PMC2789084

[97] Milosevic-Stevanovic J Krstic M Radovic-Janosevic D: Number of decidual natural killer cells & macrophages in pre-eclampsia. *Indian J Med Res.* 2016;144(6):823–30. 10.4103/ijmr.IJMR_776_15 28474619 PMC5433275

[98] Eide IP Rolfseng T Isaksen CV: Serious foetal growth restriction is associated with reduced proportions of natural killer cells in decidua basalis. *Virchows Arch.* 2006;448(3):269–76. 10.1007/s00428-005-0107-z 16328353

[99] Sánchez-Rodríguez EN Nava-Salazar S Mendoza-Rodríguez CA: Persistence of decidual NK cells and KIR genotypes in healthy pregnant and preeclamptic women: a case-control study in the third trimester of gestation. *Reprod Biol Endocrinol.* 2011;9:8. 10.1186/1477-7827-9-8 21247496 PMC3034672

[100] Stallmach T Hebisch G Orban P: Aberrant positioning of trophoblast and lymphocytes in the feto-maternal interface with pre-eclampsia. *Virchows Arch.* 1999;434(3):207–11. 10.1007/s004280050329 10190299

[101] Akhlaq M Nagi AH Yousaf AW: Placental morphology in pre-eclampsia and eclampsia and the likely role of NK cells. *Indian J Pathol Microbiol.* 2012;55(1):17–21. 10.4103/0377-4929.94848 22499294

[102] Velauthar L Plana MN Kalidindi M: First-trimester uterine artery Doppler and adverse pregnancy outcome: a meta-analysis involving 55,974 women. *Ultrasound Obstet Gynecol.* 2014;43(5):500–7. 10.1002/uog.13275 24339044

[103] Fraser R Whitley GS Thilaganathan B: Decidual natural killer cells regulate vessel stability: implications for impaired spiral artery remodelling. *J Reprod Immunol.* 2015;110:54–60. 10.1016/j.jri.2015.04.003 26004035 PMC4502446

[104] Lash GE Pitman H Morgan HL: Decidual macrophages: key regulators of vascular remodeling in human pregnancy. *J Leukoc Biol.* 2016;100(2):315–25. 10.1189/jlb.1A0815-351R 26819320

[105] Vacca P Cantoni C Vitale M: Crosstalk between decidual NK and CD14 ^+^ myelomonocytic cells results in induction of Tregs and immunosuppression. *Proc Natl Acad Sci U S A.* 2010;107(26):11918–23. 10.1073/pnas.1001749107 20547831 PMC2900704

[106] Kämmerer U Eggert AO Kapp M: Unique appearance of proliferating antigen-presenting cells expressing DC-SIGN (CD209) in the decidua of early human pregnancy. *Am J Pathol.* 2003;162(3):887–96. 10.1016/S0002-9440(10)63884-9 12598322 PMC1868095

[107] Dietl J Hönig A Kämmerer U: Natural killer cells and dendritic cells at the human feto-maternal interface: an effective cooperation? *Placenta.* 2006;27(4–5):341–7. 10.1016/j.placenta.2005.05.001 16023204

[108] Tirado-González I Muñoz-Fernández R Prados A: Apoptotic DC-SIGN+ cells in normal human decidua. *Placenta.* 2012;33(4):257–63. 10.1016/j.placenta.2012.01.003 22261157

[109] Laskarin G Redzović A Rubesa Z: Decidual natural killer cell tuning by autologous dendritic cells. *Am J Reprod Immunol.* 2008;59(5):433–45. 10.1111/j.1600-0897.2008.00599.x 18405314

[110] Blois SM Freitag N Tirado-González I: NK cell-derived IL-10 is critical for DC-NK cell dialogue at the maternal-fetal interface. *Sci Rep.* 2017;7(1):2189. 10.1038/s41598-017-02333-8 28526846 PMC5438353

[111] Croxatto D Vacca P Canegallo F: Stromal cells from human decidua exert a strong inhibitory effect on NK cell function and dendritic cell differentiation. *PLoS One.* 2014;9(2):e89006. 10.1371/journal.pone.0089006 24586479 PMC3930605

[112] Brighton PJ Maruyama Y Fishwick K: Clearance of senescent decidual cells by uterine natural killer cells in cycling human endometrium. *eLife.* 2017;6: pii: e31274. 10.7554/eLife.31274 29227245 PMC5724991

[113] Laird SM Tuckerman EM Cork BA: A review of immune cells and molecules in women with recurrent miscarriage. *Hum Reprod Update.* 2003;9(2):163–74. 10.1093/humupd/dmg013 12751778

[114] Tuckerman E Mariee N Prakash A: Uterine natural killer cells in peri-implantation endometrium from women with repeated implantation failure after IVF. *J Reprod Immunol.* 2010;87(1–2):60–6. 10.1016/j.jri.2010.07.001 20800899

[115] Quenby S Nik H Innes B: Uterine natural killer cells and angiogenesis in recurrent reproductive failure. *Hum Reprod.* 2008;24(1):45–54. 10.1093/humrep/den348 18835875

[116] Marron K Walsh D Harrity C: Detailed endometrial immune assessment of both normal and adverse reproductive outcome populations. *J Assist Reprod Genet.* 2019;36(2):199–210. 10.1007/s10815-018-1300-8 30194617 PMC6420564

[117] van der Molen RG Schutten JH van Cranenbroek B: Menstrual blood closely resembles the uterine immune micro-environment and is clearly distinct from peripheral blood. *Hum Reprod.* 2014;29(2):303–14. 10.1093/humrep/det398 24249743

[118] Gamliel M Goldman-Wohl D Isaacson B: Trained Memory of Human Uterine NK Cells Enhances Their Function in Subsequent Pregnancies. *Immunity.* 2018;48(5):951–962.e5. 10.1016/j.immuni.2018.03.030 29768178

[119] Croxatto D Micheletti A Montaldo E: Group 3 innate lymphoid cells regulate neutrophil migration and function in human decidua. *Mucosal Immunol.* 2016;9(6):1372–83. 10.1038/mi.2016.10 26906405

[120] Vacca P Pesce S Greppi M: PD-1 is expressed by and regulates human group 3 innate lymphoid cells in human decidua. *Mucosal Immunol.* 2019;12(3):624–31. 10.1038/s41385-019-0141-9 30755717

